# Dissecting Materiel

**Published:** 1878-07-20

**Authors:** 


Page(s) missing

